# The survival benefit of adjuvant trastuzumab with or without chemotherapy in the management of small (T1mic, T1a, T1b, T1c), node negative HER2+ breast cancer

**DOI:** 10.1038/s41523-024-00652-4

**Published:** 2024-06-19

**Authors:** Kai C. C. Johnson, Ai Ni, Dionisia Quiroga, Ashley C. Pariser, Preeti K. Sudheendra, Nicole O. Williams, Sagar D. Sardesai, Mathew Cherian, Daniel G. Stover, Margaret Gatti-Mays, Bhuvaneswari Ramaswamy, Maryam Lustberg, Sachin Jhawar, Roman Skoracki, Robert Wesolowski

**Affiliations:** 1 The Ohio State University Comprehensive Cancer Center, Columbus, OH USA; 2 Division of Biostatistics, The Ohio State University College of Public Health, Columbus, OH USA; 3 Smilow Cancer Hospital, Yale Cancer Center, New Haven, CT USA

**Keywords:** Breast cancer, Breast cancer

## Abstract

There is limited data regarding the added benefit of adjuvant systemic therapy in the management of small, node-negative, HER2+ breast cancer. In a multi-institutional retrospective analysis using the American Society of Clinical Oncology CancerLinQ database, we compared survival outcomes among T1a-c N0 HER2+ patients diagnosed between 2010 to 2021 who received locoregional therapy alone or in combination with adjuvant trastuzumab (+/− chemotherapy). Primary outcomes were invasive disease-free survival (iDFS) and overall survival (OS). Of the 1,184 patients, 436 received locoregional therapy alone. We found a statistically significant improvement in iDFS (HR 0.73, *P* = 0.003) and OS (HR 0.63, *P* = 0.023) on univariate analysis with adjuvant trastuzumab with or without chemotherapy which remained statistically significant on multivariate analysis. Three-arm univariate analysis found that iDFS was significantly improved with trastuzumab monotherapy (*P* = 0.003) and combination therapy (*P* = 0.027) compared to observation. Subgroup data suggests that T1b/c tumors derive the greatest benefit.

## Introduction

The topic of whether HER2+ status in breast cancer (BC) contributes to disease recurrence and survival has long been settled, leading to the advent of HER2-targeting agents such as trastuzumab^1,2^. Slamon and colleagues most famously demonstrated the added clinical benefit of trastuzumab when combined with chemotherapy in the metastatic setting where it provided an absolute overall survival (OS) benefit of nearly 5 months (25.1 months vs. 20.3 months, *P* = 0.046) and improved the objective response rate (ORR) to 50% compared to chemotherapy alone (32%, *P* < 0.001)^3^. This was later followed by the application of trastuzumab in the adjuvant setting for high-risk, node-positive HER2+ breast cancer patients based on the results of 5 landmark trials (NSABP B-31, HERA, NCCTG N9831, BCIRG-006, and FinHER)^4–8^. Cumulatively, they found that the addition of trastuzumab improved OS and reduced the 3-year breast cancer recurrence risk by nearly half.

However, largely excluded from these studies were those deemed low risk, including patients with small (T1mic, T1a, T1b, T1c), node-negative (N0) disease. Historically, HER2 positivity has been linked to higher relapse rates, including for patients with small, stage 1 cancers^9,10^. Based on additional retrospective analyses however, 5-year invasive disease-free survival (iDFS) among HER2 + BC patients with T1a-b N0 disease has ranged anywhere from 68 to 96%^9–19^. Furthermore, the lack of a standardized adjuvant endpoint for examining recurrence risk has forced researchers to compare incompatible metrics across studies^20^.

Prospective randomized data involving this population has remained sparse over the past few decades as well. The first major study to include such patients was BCIRG-006—a phase 3 clinical trial examining the clinical outcomes of those with early HER2 + BC who received an adjuvant chemotherapy regimen of either AC-T (doxorubicin and cyclophosphamide followed by docetaxel), AC-TH (AC-T in combination with 1 year of trastuzumab), or anthracycline-free TCH (docetaxel and carboplatin in combination with 1 year of trastuzumab)^6,7^. With a primary endpoint of 5-year iDFS, the investigators were able to demonstrate considerable improvements to iDFS for those on a trastuzumab-containing regimen (AC-TH or TCH). However, given the low number of T1N0 patients enrolled, no firm conclusions could be reached for this unique patient subset.

While several single-arm studies have since taken a closer look into the management of T1N0 patients, few have attempted to differentiate whether the benefit derived from systemic therapy is due to trastuzumab therapy itself or in combination with chemotherapy^21–24^. Furthermore, whether systemic therapy itself adds benefit versus locoregional therapy alone remains unanswered in this patient population (particularly in patients with T1a and T1b tumors). This is further echoed within the National Comprehensive Cancer Network (NCCN) guidelines, where it states that for node-negative pT1a and pT1b patients, the toxicities of trastuzumab must be balanced amongst the uncertain absolute benefits of trastuzumab^25^.

Given the findings above, current evidence remains insufficient as to whether adjuvant trastuzumab provides any additional benefit for this patient population. Our study sought to help address this question by reviewing multi-institutional data for patients with small, node-negative, HER2+ breast cancer to compare clinical outcomes between those who go untreated versus those who receive adjuvant trastuzumab therapy, with or without chemotherapy.

## Results

### Patient characteristics

Medical records of 19,112 patients with HER2+ breast cancer were identified in the American Society of Clinical Oncology (ASCO) CancerLinQ database. Of these, 17,928 did not meet eligibility criteria and were excluded. Of the remaining 1184 patients who met study eligibility criteria, 169 received adjuvant trastuzumab therapy alone, 579 received a combination of trastuzumab and chemotherapy, and 436 received neither chemotherapy nor trastuzumab as part of their care (Supplemental Fig. 1). Median follow-up was 77.6 months (range 4.6–137 months) for the entire study population, 77.8 months (range 7.6–136.9 months) for untreated patients, 77.5 months (range 4.6–137 months) for patients treated with trastuzumab plus chemotherapy, and 78.5 months (range 15–136 months) for patients who received trastuzumab monotherapy. Baseline characteristics for each treatment group are provided in Table 1. Overall, the patient population was primarily post-menopausal Caucasian women with intermediate- to high-grade tumors. Fifty-two percent of patients had pT1c tumors, while 27.4% and 17.1% of patients had pT1b and pT1a tumors, respectively. Approximately 54.9% of patients had hormone receptor-positive (HR + ) disease, and of those patients, 77.5% received endocrine therapy with or without ovarian suppression where applicable. For patients who received adjuvant trastuzumab, we found that the mean number of cycles administered was 13.2 (standard deviation (SD) 4.79). The baseline characteristics were generally similar between the untreated, combination therapy, and trastuzumab alone groups. In terms of chemotherapy administered, 218 patients in the combination arm (37.7%) received adjuvant taxane chemotherapy plus trastuzumab alone (TH), whereas the remainder received a multiagent chemotherapy regimen.

**Table 1:** Summary of baseline characteristics across different treatment groups

	Trastuzumab alone (*n* = 169)	Combination therapy (*n* = 579)	Untreated (*n* = 436)	Overall (*n* = 1184)
Age				
Mean (SD)	58.7 (12.2)	58.3 (11.3)	63.1 (12.8)	60.1 (12.2)
Median [Min, Max]	59.1 [26.8, 85.2]	58.8 [18.9, 84.9]	62.9 [25.9, 95.4]	60.4 [18.9, 95.4]
<50	41 (24.3%)	138 (23.8%)	71 (16.3%)	250 (21.1%)
≥50	128 (75.7%)	441 (76.2%)	364 (83.5%)	933 (78.8%)
BMI				
Mean (SD)	28.3 (7.45)	28.8 (6.24)	28.5 (7.18)	28.6 (6.79)
Median [Min, Max]	26.8 [17.4, 65.4]	27.9 [16.7, 52.0]	27.2 [10.9, 61.4]	27.5 [10.9, 65.4]
<25	44 (26.0%)	121 (20.9%)	113 (25.9%)	278 (23.5%)
≥30	36 (21.3%)	151 (26.1%)	111 (25.5%)	298 (25.2%)
≥25– < 30	37 (21.9%)	117 (20.2%)	105 (24.1%)	259 (21.9%)
Missing	52 (30.8%)	190 (32.8%)	107 (24.5%)	349 (29.5%)
Race				
Black	20 (11.8%)	36 (6.2%)	34 (7.8%)	90 (7.6%)
White	109 (64.5%	361 (62.3%)	268 (61.5%)	738 (62.3%)
Other	40 (23.7%)	182 (31.4%)	134 (30.7%)	355 (30.1%)
Ethnicity				
Hispanic or Latino	10 (5.9%)	25 (4.3%)	15 (3.4%)	50 (4.2%)
Neither	144 (85.2%)	496 (85.7%)	342 (78.4%)	982 (82.9%)
T stage				
T1a	41 (24.3%)	63 (10.9%)	98 (22.5%)	202 (17.1%
T1b	33 (19.5%)	157 (27.1%)	135 (31.0%)	325 (27.4%)
T1c	87 (61.5%)	350 (60.4%)	178 (40.8%)	615 (51.9%)
T1mic	2 (1.2%)	0 (0.0%)	12 (2.8%)	14 (1.2%)
Unknown subtype	6 (3.6%)	9 (1.6%)	13 (3.0%)	28 (2.4%)
Tumor grade				
Grade 1	9 (5.3%)	44 (7.6%)	73 (16.7%)	126 (10.6%)
Grade 2	73 (43.2%)	225 (38.9%)	196 (45.0%)	494 (41.7%)
Grade 3	82 (48.5%)	291 (50.3%)	154 (35.3%)	527 (44.5%)
Unknown grade	5 (3.0%)	19 (3.3%)	13 (3.0%)	37 (3.1%)
Smoking history				
Never smoker	102 (60.4%)	324 (56.0%)	252 (57.8%)	678 (57.3%)
Current or former	60 (35.5%)	192 (33.2%)	139 (31.9%)	391 (33.0%)
BRCA status				
Positive	2 (1.2%)	18 (3.1%)	11 (2.5%)	31 (2.6%)
Negative	39 (23.1%)	148 (25.6%)	69 (15.8%)	256 (21.6%)
HR status				
Positive	83 (49.1%)	308 (53.2%)	259 (59.4%)	650 (54.9%)
Negative	82 (48.5%)	264 (45.6%)	173 (39.7%)	519 (43.8%)
Histology				
IDC	103 (60.9%)	346 (59.8%)	281 (64.4%)	730 (61.7%)
ILC	55 (32.5%)	207 (35.8%)	129 (29.6%)	391 (33.0%)
Number of trastuzumab cycles				
Median [Min, Max]	12.0 [1.00, 18.0]	13.0 [1.00, 35.0]	N/A	13.0 [1.00, 35.0]
**ECOG**				
0	53 (31.4%)	210 (36.3%)	103 (23.6%)	366 (30.9%)
1	20 (11.8%)	70 (12.1%)	45 (10.3%)	135 (11.4%)
≥2	4 (2.4%)	6 (1.0%)	6 (1.4%)	16 (1.4%)
Endocrine therapy for HR+ patients				
No	16/83 (19.3%)	52/308 (16.9%)	78/259 (30.1%)	146/650 (22.5%)
Yes	67/83 (80.7%)	256/308 (83.1%)	181/259 (69.9%)	504/650 (77.5%)
Geographic region				
Midwest	25 (14.8%)	94 (16.2%)	75 (17.2%)	194 (16.4%)
Northeastern	9 (5.3%)	69 (11.9%)	45 (10.3%)	123 (10.4%)
Southern	79 (46.7%)	281 (48.5%)	167 (38.3%)	527 (44.5%)
Western	53 (31.4%)	126 (21.8%)	141 (32.3%)	320 (27.0%)
Follow-up				
Median follow-up (months)	78.5	77.5	77.8	77.6

*BMI* body mass index, *ECOG* Eastern Cooperative Oncology Group, *IDC* invasive ductal carcinoma, *ILC* invasive lobular carcinoma, *Min* minimum, *Max* maximum, *N/A* not applicable, *SD* standard deviation.

### Invasive disease-free survival

For the two-arm analysis comparing those who received trastuzumab with or without chemotherapy to those who received neither (labeled as “untreated” for simplicity), the 3-year and 5-year iDFS rates were 85.4% and 82.9% for the treated arm versus 80.9% and 76.1% in the untreated arm (Table 2).

**Table 2:** Summary of 3-year and 5-year iDFS and OS within two-arm and three-arm comparisons

	3-year iDFS	5-year iDFS	3-year OS	5-year OS
Untreated	80.9% 95% CI 77.3–84.7%	76.1% 95% CI 72.1–80.3%	97.5% 95% CI 96.0–99.0%	94.0% 95% CI 91.7–96.4%
Trastuzumab +/− chemotherapy	85.4% 95% CI 83.0–88.0%	82.9% 95% CI 80.2–85.6%	97.8% 95% CI 96.7–98.8%	95.6% 95% CI 94.1–97.1%
Trastuzumab monotherapy	88.1% 95% CI 83.4–93.2%	86.8% 95% CI 81.8–92.1%	97.0% 95% CI 94.5–99.6%	96.4% 95% CI 93.6–99.3%
Combination therapy	84.4% 95% CI 81.5–87.4%	81.6% 95% CI 78.5–84.9%	98.0% 95% CI 96.9–99.1%	95.4% 95% CI 93.7–97.1%

*CI* confidence interval, *iDFS* invasive disease-free survival, *OS* overall survival.

**Fig. 1:**
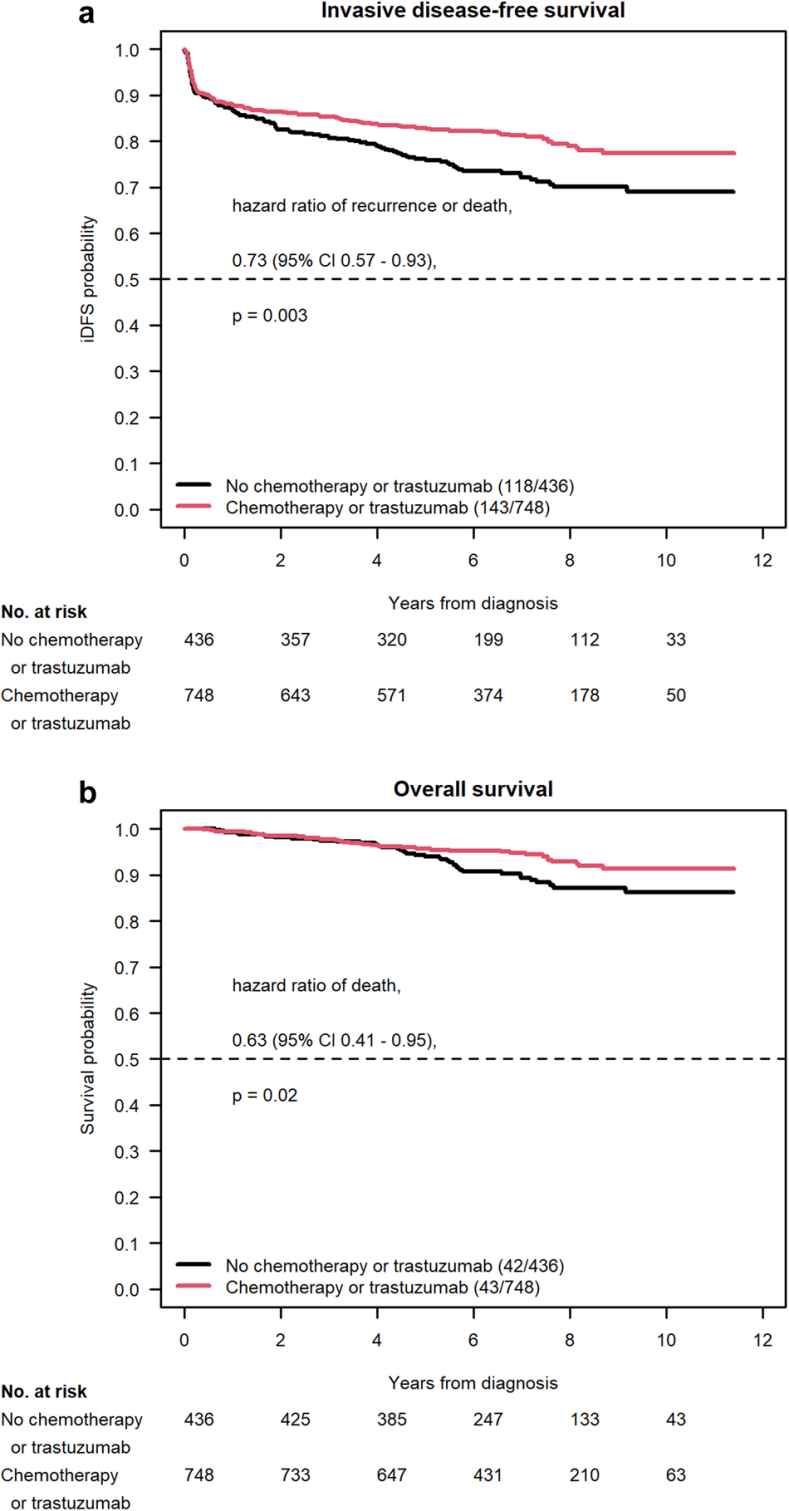
Survival outcomes with or without systemic therapy for T1N0 HER2+ breast cancer. Univariate analysis of iDFS (**a**) and OS (**b**) in our two-group comparison of adjuvant trastuzumab with or without systemic chemotherapy versus locoregional therapy alone.

We found that the primary endpoint of improved iDFS was met on univariate analysis (HR 0.73, 95% CI 0.57–0.93, *P* = 0.003) (Fig. 1 and Table 3). Furthermore, on multivariate analysis, improvements in iDFS (HR 0.70, 95% CI 0.54–0.91, *P* = 0.009) remained statistically significant.

**Table 3:** Summary of iDFS and OS using both univariate and multivariable analyses in the 2-arm and 3-arm comparisons

	iDFS	OS
Univariate analysis		
Trastuzumab +/− chemotherapy vs. untreated	HR 0.73 95% CI 0.57–0.93 *P* = 0.003**	HR 0.63 95% CI 0.41–0.95 *P* = 0.023**
Trastuzumab vs. untreated	HR 0.51 95% CI 0.33–0.79 *P* = 0.003**	HR 0.49 95% CI 0.23–1.05 *P* = 0.066
Combination vs. untreated	HR 0.75 95% CI 0.58–0.97 *P* = 0.027**	HR 0.64 95% CI 0.41–1.00 *P* = 0.053
Combination vs. trastuzumab	HR 1.46 95% CI 0.95–2.24 *P* = 0.087	HR 1.30 95% CI 0.60–2.81 *P* = 0.99
Multivariate analysis		
Trastuzumab +/− chemotherapy vs. untreated	HR 0.70 95% CI 0.54–0.91 *P* = 0.009**	HR 0.63 95% CI 0.40–0.99 *P* = 0.047**
Trastuzumab vs. untreated	HR 0.56 95% CI 0.36–0.88 *P* = 0.011**	*P* = 0.139
Combination vs. untreated	HR 0.75 95% CI 0.57–0.98 *P* = 0.038**	
Combination vs. trastuzumab	HR 1.33 95% CI 0.85–2.06 *P* = 0.21	

*CI* confidence interval, *HR* hazard ratio, *iDFS* invasive disease-free survival, *OS* overall survival.

**Denotes statistical significance between comparator groups. However, *P* values were not adjusted for multiple comparisons. Multivariate analysis using a Cox proportional hazard model was used to control for the following factors: age, race, ethnicity, BMI, hormone receptor status, tumor grade, histology type, T1 substage, BRCA mutation status, geographical region, functional status, and smoking history.

**Fig. 2:**
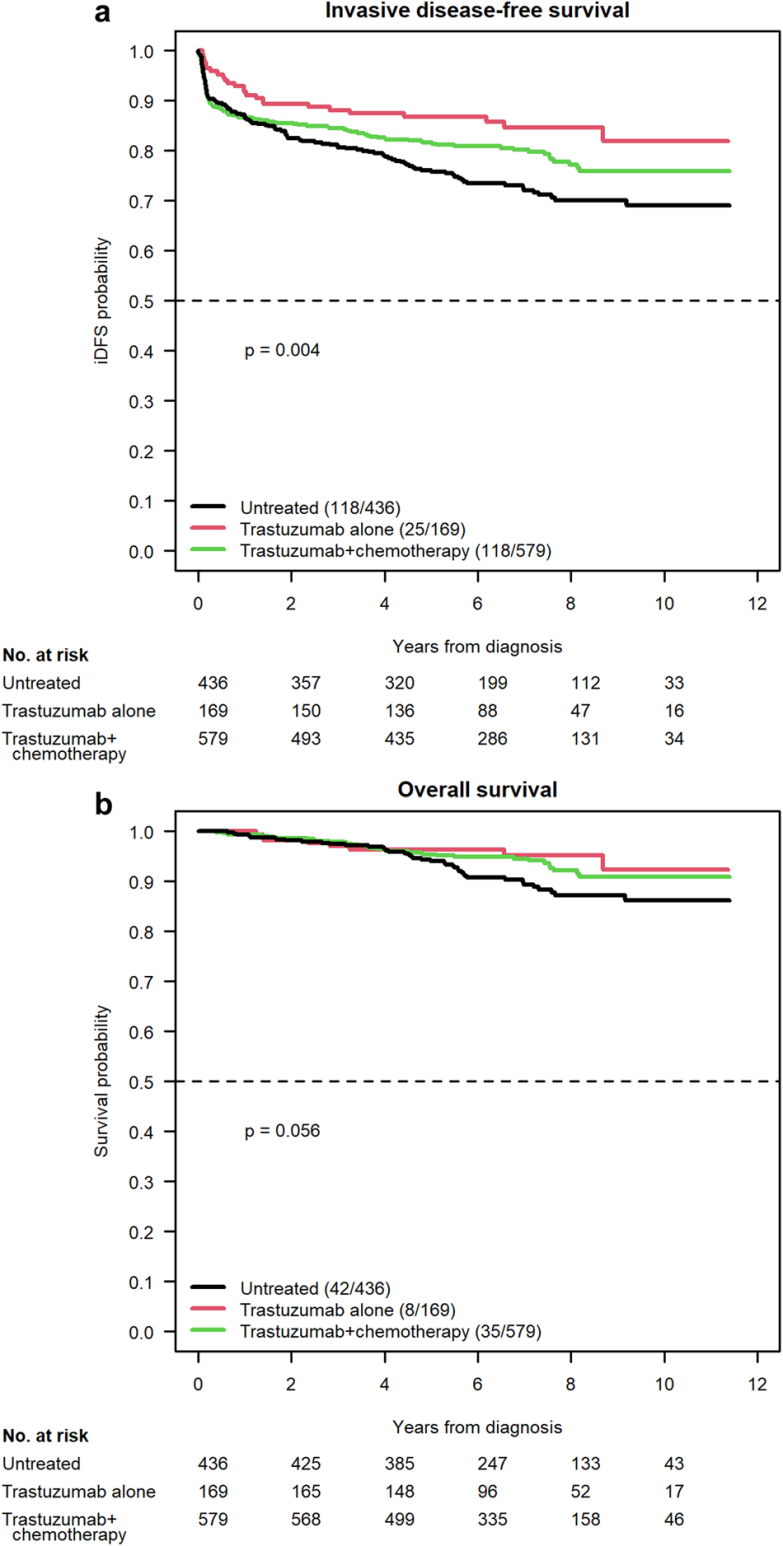
Survival outcomes for T1N0 HER2+ breast cancer following locoregional therapy alone versus trastuzumab monotherapy versus combination therapy. Univariate analysis of iDFS (**a**) and OS (**b**) in our three-group comparison of locoregional therapy alone versus trastuzumab monotherapy versus chemotherapy with trastuzumab.

We also performed a three-arm analysis, further breaking down clinical outcomes based on whether patients were untreated, received trastuzumab monotherapy, or received a combination of trastuzumab and chemotherapy. Univariate findings for iDFS and OS are illustrated in Fig. 2. In terms of iDFS, a significant improvement was seen in those who received combination therapy versus those who went untreated, both on univariate analysis (HR 0.75, 95% CI 0.58–0.9647, *P* = 0.027) and multivariate analysis (HR 0.75, 95% CI 0.57–0.98, *P* = 0.038). Similarly, those who received trastuzumab alone had a significant improvement in iDFS compared to untreated patients on both univariate (HR 0.51, 95% CI 0.33–0.79, *P* = 0.003) and multivariate (HR 0.56, 95% CI 0.36–0.88, *P* = 0.011) analysis. When comparing combination therapy to trastuzumab monotherapy, no clear difference was noted on either analysis (univariate: HR 1.46, 95% CI 0.95–2.24, *P* = 0.087; multivariate: HR 1.33, 95% CI 0.85–2.06, *P* = 0.21).

**Fig. 3:**
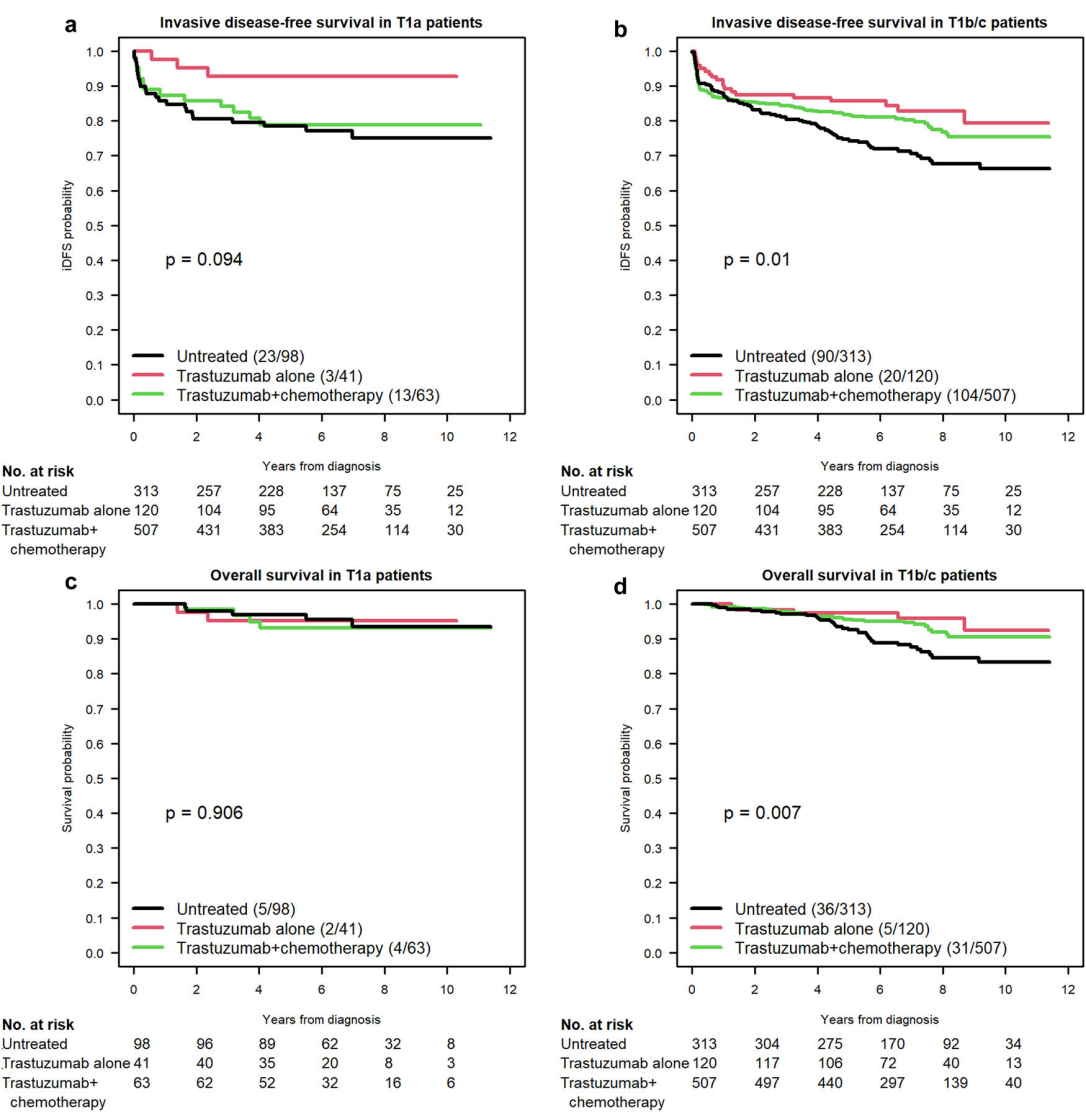
Survival outcomes for T1N0 HER2+ breast cancer based on T1 subgroup following locoregional therapy alone versus trastuzumab monotherapy versus combination therapy. Subgroup analysis of iDFS (**a**, **b**) and OS (**c**, **d**) within the 3-arm treatment comparison with an overall test of treatment effect based on T1a (**a**, **c**) and T1b/c (**b**, **d**) tumor sub-categorization.

Within the three-arm analysis comparing iDFS between patients receiving trastuzumab monotherapy, combination therapy, and locoregional therapy alone, we performed both a subgroup univariate and multivariate analysis of iDFS among those with T1a and those with T1b/c (Fig. 3 and Supplementary Table 1). For T1a patients, the univariate hazard ratio of iDFS between the trastuzumab monotherapy group and the untreated group was 0.28 (95% CI 0.09–0.95, *P* = 0.041) and 0.23 (95% CI 0.06–0.84, *P* = 0.026) on multivariate analysis in favor of adjuvant systemic therapy. Between the combination therapy T1a group and the untreated T1a group, the univariate hazard ratio for iDFS was 0.88 (95% CI 0.44–1.73, *P* = 0.707), whereas the multivariate hazard ratio was 0.78 (95% CI 0.35–1.73, *P* = 0.544). With the two-arm comparison, the subgroup analysis comparing trastuzumab with or without chemotherapy to locoregional therapy alone in T1a patients noted a univariate hazard ratio of 0.63 (95% CI 0.33–1.19, *P* = 0.154) and a multivariate hazard ratio of 0.53 (95% CI 0.26–1.09, *P* = 0.084) for iDFS.

We performed the same iDFS subgroup analysis for those with either T1b or T1c tumors. With the three-arm comparison, the univariate hazard ratio for iDFS when comparing trastuzumab monotherapy to locoregional therapy alone was 0.55 (95% CI 0.34–0.89, *P* = 0.014) and the multivariate hazard ratio was 0.62 (95% CI 0.37–1.02, *P* = 0.061). For combination therapy versus untreated patients with T1b/T1c disease, the univariate hazard ratio was 0.71 (95% CI 0.53–0.94, *P* = 0.016) and the multivariate hazard ratio was 0.70 (95% CI 0.52–0.95, *P* = 0.023). Finally, for the two-arm comparison of T1b/T1c patients (*n* = 940, *n* of event = 214) who received trastuzumab with or without chemotherapy versus untreated patients, the univariate hazard ratio was 0.68 (95% CI 0.51–0.89, *P* = 0.004) and the multivariate hazard ratio was 0.69 (95% CI 0.52–0.92, *P* = 0.012).

Within the Supplementary Appendix, a separate subgroup analysis comparing iDFS among T1a vs. T1b vs. T1c cohorts was examined. This included both the two-group and three-group analyses to provide more granular data between subgroups. The results are summarized within Supplemental Figs. 2 and 3 and Supplemental Table 2.

### Overall survival

A significant improvement in OS was noted on the two-arm univariate analysis comparing treated patients to untreated participants (HR 0.63, 95% CI 0.41–0.95, *P* = 0.023). The 3-year and 5-year OS rates were 97.8% and 95.6% in the treated arm versus 97.5% and 94.0% in the untreated arm, respectively. On multivariate analysis of OS, significant improvements were still seen (HR 0.63, 95% CI 0.40–0.99, *P* = 0.047). Hormone receptor status initially caused failure of the multivariate regression model, but this was solved by excluding patients with unknown hormone receptor status from the multivariate analysis, which accounted for 15 patients total.

When performing univariate analysis for OS between patients treated with trastuzumab monotherapy, combination with chemotherapy, and those receiving no treatment, there was a marginal difference among the 3 groups (*P* = 0.056). A statistically non-significant trend toward improvement was noted in both the combination versus untreated comparison (HR 0.64, 95% CI 0.41–1.00, *P* = 0.053) and the trastuzumab monotherapy versus untreated comparison (HR 0.49, 95% CI 0.23–1.05, *P* = 0.066). On multivariate analysis of OS using this three-arm comparison, no significant association was noted overall (*P* = 0.139); therefore, the pairwise test results were not examined. Similar to before, hormone receptor status caused regression model failure, which was solved again by excluding the 15 patients with unknown hormone receptor status from the multivariate analysis of OS.

Similar to before with iDFS, we performed a univariate subgroup analysis for T1a patients (*n* = 202, number of events = 11) and T1b/T1c patients (*n* = 940, number of event = 72), both for the three-arm and two-arm comparisons. However, the multivariate models for OS could not be performed due to small event rates. When reviewing T1a patients who received trastuzumab with or without chemotherapy versus those untreated, the univariate OS hazard ratio was 1.21 (95% CI 0.37–3.96, *P* = 0.756), whereas for T1b/T1c patients, it was 0.5 (95% CI 0.31–0.79, *P* = 0.002). For the three-arm comparison, T1a patients who received trastuzumab monotherapy versus observation had a univariate OS hazard ratio of 1.02 (95% CI 0.2–5.27, *P* = 0.981), whereas for T1b/T1c patients, it was 0.35 (95% CI 0.14–0.90, *P* = 0.027). For combination therapy versus observation, the univariate OS hazard ratio for T1a patients was 1.33 (95% CI 0.36–4.95, *P* = 0.673) and for T1b/T1c patients it was 0.53 (95% CI 0.33–0.86, *P* = 0.01). Lastly, for combination therapy versus trastuzumab monotherapy, the univariate hazard ratio was 1.3 (95% CI 0.24–7.11, *P* = 0.761) in the T1a cohort and 1.53 (95% CI 0.6–3.94, *P* = 0.375) in the T1b/T1c cohort. The overall test on treatment effect was insignificant for T1a patients (*P* = 0.906) but highly significant for T1b/T1c patients (*P* = 0.007).

Similar to our iDFS comparison, a separate subgroup analysis comparing OS among T1a vs. T1b vs. T1c cohorts was examined. This was performed using both the two-group and three-group datasets, but only via univariate analysis due to low event rates. The results are summarized within Supplemental Figs. 4 and 5 and Supplemental Table 2.

## Discussion

This non-randomized retrospective study found that the use of adjuvant trastuzumab therapy was associated with a significant improvement in iDFS, whether used in combination with chemotherapy or alone as monotherapy. Results were generally similar on univariate and multivariate analysis. However, while patients with T1b/c breast cancer who received adjuvant systemic treatment with trastuzumab with or without chemotherapy experienced statistically significant iDFS and OS survival benefit compared to untreated patients, this was less evident with the T1a subgroup.

As mentioned earlier, only a handful of prospective studies have examined how HER2-directed therapies impact recurrence risk for this unique patient population. This is due to multiple factors, including the sheer size of patients needed in order to meaningfully detect a change in outcomes given the favorable prognosis of stage 1 breast cancer. Most of these prospective studies involved not only small node-negative patients, but also those with large tumor sizes, positive nodal status, and high-risk features^4–8^. As a result, we often see an overall improvement in recurrence risk within the primary data, but little remains that’s specific to this subset of patients outside of post-hoc analyses. We previously published a review that summarized the various retrospective and prospective studies that include this patient population^16^. Most notable are the newer propensity-matched analyses, such as one by Parsons et al., which examined data from the National Cancer Database (NCDB) from 5720 T1N0 HER + BC patients with untreated disease, then propensity-matched them 1:1 to 15,428 T1N0 HER2 + BC patients who received systemic therapy with or without anti-HER2 agents^17^. While improvements in 5-year OS were seen in T1b (97.1% vs. 92.3%, *P* = 0.0016) and T1c patients (95.9% vs. 91.9%, *P* = 0.0002) when systemic therapy was received versus locoregional therapy alone, T1mic patients fared much worse with systemic therapy versus observation (89.1% vs. 99.1% respectively, *P* = 0.0006) with a trend toward harm seen in T1a patients (95.4% vs. 96.9%, *P* = 0.059) as well. Given these findings, this study highlights the potential harms of using a one-size-fits-all approach to managing this patient population. These findings were validated by another NCDB study by Cao et al. where T1aN0 HER2 + BC patients who received locoregional therapy alone (*n* = 4026) were propensity-matched 1:1 to those who received the addition of systemic therapy (*n* = 4922), with the result demonstrating no significant improvement in OS with systemic therapy versus locoregional therapy alone (HR 1.613, *P* = 0.107)^18^.

While the sample size was remarkable for both propensity-matched studies, the NCDB provided authors no clear distinction between chemotherapy and anti-HER2 therapy, limiting its ability to answer the question of whether chemotherapy or anti-HER2 therapy was the driver of benefit in the T1b and T1c populations. Cao and colleagues performed a separate study using NCDB data in T1N0 patients to help differentiate benefits seen from combination therapy versus anti-HER2 monotherapy alone, but it is unclear if similar issues arose, and the results were inconclusive when multivariable Cox proportional hazard regression was applied^19^. Overall, our study found that anti-HER2 monotherapy was similarly associated with an improvement in both iDFS and OS compared to combination therapy, with both systemic options showing efficacy over locoregional therapy alone. While this finding itself does not justify the use of trastuzumab monotherapy across all T1N0 HER2 + BC patients (given the limitations of retrospective analysis), it does warrant further investigation into how chemotherapy-free regimens could be applied to risk-stratified patients within this population. Such efforts have been attempted in the neoadjuvant setting, as seen with trials such as PerELISA, PAMELA, and CALGB 40601 where intrinsic subtyping helped predict likelihood of pathologic complete response (pCR) rates^26–28^. Novel genomic assays, such as HER2DX, also appear to reliably predict pCR and newer risk scores have been shown to closely correlate to clinical outcomes when retrospectively applied to updated data from the APT trial^29,30^. More recently, trials like PHERGain have demonstrated the feasibility of chemotherapy-free anti-HER2 regimens when using fluorodeoxyglucose [^18^F] positron emission tomography for the monitoring of treatment response to help guide treatment escalation where needed^31^.

Major limitations to our study include its inherent retrospective design. Despite our best efforts to match clinical and intrinsic factors when performing multivariable analysis, selection bias plays a role in why certain patients receive systemic therapy as compared to patients who go “untreated.” This in turn can impact the clinical outcomes we see. We attempted to correct for this by performing multivariate analysis. However, it is not possible to correct for all confounding variables, especially selection bias. We were also unable to analyze distant disease-free survival separately, which would be of value given that overall survival comparisons in non-randomized low risk populations can result in inaccurate reflections of breast cancer related deaths when they may otherwise be attributed to noncancerous conditions, further propagating selection bias. Furthermore, while we attempted to match up multiple variables for each patient, not every data point had a known answer, such as BRCA status, body mass index (BMI), or functional status. We also did not have access to information pertaining to margin status or multifocal disease. Finally, while our sample size was large compared to historic retrospective studies involving T1N0 HER2 + BC patients, it lacked data on the T1mic substage (*n* = 14), so no meaningful conclusions could be drawn from this study population.

Our findings suggest that adjuvant trastuzumab, with or without chemotherapy, is associated with an added benefit among patients with small, node-negative HER2+ breast cancer through significant improvements in iDFS. A clear benefit was observed for patients with T1b/T1c tumors in terms of both iDFS and OS whereas in those with T1a tumors, this benefit was not seen. Though the results are limited by biases inherent to retrospective analysis, this study further adds to the growing evidence surrounding the benefit of adjuvant trastuzumab in this patient population, including when given as monotherapy as shown in the three-arm analysis. Further stratification efforts are needed to identify low-risk individuals suitable for such de-escalated therapies. Finally, given the unlikeliness that observation alone would be provided as a treatment modality in a randomized prospective study, more large-scale retrospective data is needed to provide us with better insight into baseline recurrence risk and the net benefit of systemic therapies when accounting for risks of serious adverse events.

## Methods

### Study design

Using the ASCO CancerLinQ database, we performed a retrospective analysis focused on clinical data from small, node-negative HER2 + BC patients diagnosed between 2010 to 2021. This national patient data repository does not contain patient-identifying information and was therefore exempt from Institutional Review Board approval. Specifically, the inclusion criteria comprised patients with pathologically confirmed node-negative breast cancer (pN0), tumor sizes ≤2.0 cm (pT1), confirmation of HER2+ status through immunohistochemistry and/or in situ hybridization methodology in accordance with the ASCO/College of American Pathologists (CAP) guidelines that were used at the time of the diagnosis, available medication-specific administration data, and adequate follow-up data for the evaluation of disease recurrence status and OS status (Supplemental Fig. 1). Those who received neoadjuvant systemic therapy were excluded from the study.

This national database includes deidentified patient data stored from a diverse mix of academic and community oncology centers found across all 50 US states to provide a comprehensive overview of real-world practices and outcomes. Data pertaining to diagnoses, laboratory tests, genetic data, pathologic findings, locoregional therapy, systemic therapies, imaging, adverse events, and clinical outcomes is stored within CancerLinQ. Treatment and testing data is time stamped, allowing for accurate timelines to be created surrounding individual patient care. Furthermore, specific infusion data surrounding treatment cycle dates is provided to assist in confirming receipt of medications.

### Statistical methods

The primary outcomes were iDFS and OS, based on definitions used within the STEEP 2.0 guidelines for the standardization of adjuvant endpoints^20^. The adjuvant endpoint iDFS is defined as the time from diagnosis until the time of invasive recurrence of any type or death from any cause. OS was defined as the time from diagnosis until the time of death from any cause. We defined “untreated” patients as those who received locoregional therapy alone, though use of adjuvant endocrine therapy was allowed where applicable. We performed a univariate analysis using log-rank testing to compare survival outcomes between those who received adjuvant trastuzumab, with or without chemotherapy, to those who did not. We estimated 3-year and 5-year iDFS and OS using the Kaplan–Meier method. We then performed a multivariate analysis using a Cox proportional hazard model to control for factors including age, race, ethnicity, BMI, hormone receptor status, tumor grade, histology type, T1 substage, BRCA mutation status, region, functional status, and smoking history. Additionally, we performed a three-arm univariate analysis in order to compare outcomes between untreated patients, patients on trastuzumab monotherapy, and patients who received a combination of trastuzumab and chemotherapy. Finally, we conducted prespecified subgroup analysis by T stage by testing the interaction between treatment and T stage (T1a vs. T1b vs. T1c) as well as estimating the hazard ratio of treatment within each T stage. The subgroup analysis was conducted using a multivariate model for iDFS and a univariate model for OS due to the small number of deaths. All statistical tests were two-sided at a 0.05 significance level. The subgroup analyses were exploratory and therefore no multiple comparison adjustment was conducted. Statistical analysis was conducted in R, version 4.1.3. For our multiple comparisons, *P* values were note adjusted given the scope of this moderate-sized retrospective analysis.

### Reporting summary

Further information on research design is available in the Nature Research Reporting Summary linked to this article.

### Supplementary information

Supplementary Information

Reporting Summary

## Data Availability

The datasets used and/or analyzed during the current study are available from the corresponding author upon reasonable request.

## Code availability

The code is available in the CancerLinQ database for anyone who has a valid subscription to the database. Data extraction and analysis code is available upon request.

## References

[1] Slamon DJ (1987). Human breast cancer: correlation of relapse and survival with amplification of the HER-2/neu oncogene. Science.

[2] Moasser MM (2007). The oncogene HER2: its signaling and transforming functions and its role in human cancer pathogenesis. Oncogene.

[3] Slamon DJ (2001). Use of chemotherapy plus a monoclonal antibody against HER2 for metastatic breast cancer that overexpresses HER2. New Engl. J. Med..

[4] Romond EH (2005). Trastuzumab plus adjuvant chemotherapy for operable HER2-positive breast cancer. New Engl. J. Med..

[5] Piccart-Gebhart MJ (2005). Herceptin adjuvant (HERA) trial study team. trastuzumab after adjuvant chemotherapy in HER2-positive breast cancer. New Engl. J. Med..

[6] Slamon D (2005). Phase III randomized trial comparing doxorubicin and cyclophosphamide followed by docetaxel (AC-T) with doxorubicin and cyclophosphamide followed by docetaxel and trastuzumab (AC-TH) with docetaxel, carboplatin and trastuzumab (TCH) in HER2 positive early breast cancer patients: BCIRG 006 study. Breast Cancer Res. Treat..

[7] Slamon D (2011). Adjuvant trastuzumab in HER2-positive breast cancer. New Engl. J. Med..

[8] Joensuu H (2009). Fluorouracil, epirubicin, and cyclophosphamide with either docetaxel or vinorelbine, with or without trastuzumab, as adjuvant treatments of breast cancer: final results of the FinHer trial. J. Clin. Oncol..

[9] Gonzalez-Angulo AM (2009). High risk of recurrence for patients with breast cancer who have human epidermal growth factor receptor 2-positive, node-negative tumors 1 cm or smaller. J. Clin. Oncol..

[10] Curigliano G (2009). Clinical relevance of HER2 overexpression/amplification in patients with small tumor size and node-negative breast cancer. J. Clin. Oncol..

[11] Vaz-Luis I (2014). Outcomes by tumor subtype and treatment pattern in women with small, node-negative breast cancer: a multi-institutional study. J. Clin. Oncol..

[12] Fehrenbacher L (2014). Distant invasive breast cancer recurrence risk in human epidermal growth factor receptor 2-positive T1a and T1b node-negative localized breast cancer diagnosed from 2000 to 2006: a cohort from an integrated health care delivery system. J. Clin. Oncol..

[13] Si J (2020). Multiple microinvasion foci in ductal carcinoma in situ is associated with an increased risk of recurrence and worse survival outcome. Front. Oncol..

[14] McArthur HL (2011). Adjuvant trastuzumab with chemotherapy is effective in women with small, node-negative, HER2-positive breast cancer. Cancer.

[15] Kiess AP (2012). Adjuvant trastuzumab reduces locoregional recurrence in women who receive breast-conservation therapy for lymph node-negative, human epidermal growth factor receptor 2-positive breast cancer. Cancer.

[16] Johnson KC, Quiroga D, Sudheendra P, Wesolowski R (2022). Treatment of small (T1mic, T1a, and T1b) node-negative HER2+ breast cancer—a review of current evidence for and against the use of anti-HER2 treatment regimens. Expert Rev. Anticancer Ther..

[17] Parsons BM, Uprety D, Smith AL, Borgert AJ, Dietrich LL (2018). A US registry-based assessment of use and impact of chemotherapy in stage I HER2-positive breast cancer. J. Natl. Compr. Cancer Netw. JNCCN.

[18] Cao L (2022). A comparison of local therapy alone with local plus systemic therapy for stage I pT1aN0M0 HER2+ breast cancer: a National Cancer Database analysis. Cancer.

[19] Cao L (2022). Adjuvant trastuzumab with or without chemotherapy in stage 1 pT1N0 HER2+ breast cancer: a National Cancer Database analysis. Breast Cancer Res. Treat..

[20] Tolaney SM (2021). Updated standardized definitions for efficacy end points (STEEP) in adjuvant breast cancer clinical trials: STEEP version 2.0. J. Clin. Oncol..

[21] Jones SE (2013). Adjuvant docetaxel and cyclophosphamide plus trastuzumab in patients with HER2-amplified early stage breast cancer: a single-group, open-label, phase 2 study. Lancet Oncol..

[22] Tolaney SM (2015). Adjuvant paclitaxel and trastuzumab for node-negative, HER2-positive breast cancer. New Engl. J. Med..

[23] Bellon JR (2019). Local-regional recurrence in women with small node-negative, HER2-positive breast cancer: results from a prospective multi-institutional study (the APT trial). Breast Cancer Res. Treat..

[24] Tolaney SM (2021). Adjuvant trastuzumab emtansine versus paclitaxel in combination with trastuzumab for stage I HER2-positive breast cancer (ATEMPT): a randomized clinical trial. J. Clin. Oncol..

[25] Gradishar, W. et al. *NCCN Guidelines: Breast Cancer (version 4.2023)* (National Comprehensive Cancer Network (NCCN), 2023). 10.6004/jnccn.2023.0031 37308117

[26] Prat A (2020). HER2-enriched subtype and ERBB2 expression in HER2-positive breast cancer treated with dual HER2 blockade. *JNCI*. J. Natl. Cancer Inst..

[27] Llombart-Cussac A (2017). HER2-enriched subtype as a predictor of pathological complete response following trastuzumab and lapatinib without chemotherapy in early-stage HER2-positive breast cancer (PAMELA): an open-label, single-group, multicentre, phase 2 trial. Lancet Oncol..

[28] Fernandez-Martinez A (2020). Survival, pathologic response, and genomics in CALGB 40601 (alliance), a neoadjuvant phase III trial of paclitaxel-trastuzumab with or without lapatinib in HER2-positive breast cancer. J. Clin. Oncol..

[29] Guarneri V (2022). HER2DX genomic test in HER2-positive/hormone receptor-positive breast cancer treated with neoadjuvant trastuzumab and pertuzumab: a correlative analysis from the PerELISA trial. EBioMedicine.

[30] Tolaney SM (2023). Adjuvant paclitaxel and trastuzumab for node-negative, HER2-positive breast cancer: final 10-year analysis of the open-label, single-arm, phase 2 APT trial. Lancet Oncol..

[31] Pérez-García JM (2022). Trastuzumab and pertuzumab without chemotherapy in early-stage HER2+ breast cancer: a plain language summary of the PHERGain study. Future Oncol..

