# Supplementary material for: The survival benefit of adjuvant trastuzumab with or without chemotherapy in the management of small (T1mic, T1a, T1b, T1c), node negative HER2+ breast cancer
Source: NPJ Breast Cancer. 2024 Jun 19;10:49. doi: 10.1038/s41523-024-00652-4 (PMC11187074; doi:10.1038/s41523-024-00652-4)
Supplement: Supplementary file 1 — Supplementary Information [file 41523_2024_652_MOESM1_ESM.pdf]

Supplementary Information

Supplemental Table 1. Summary of iDFS and OS within our two- and three-arm comparisons when classified by T1a versus T1b/c substage

	T1a iDFS	T1b/c iDFS	T1a OS	T1b/c OS
**Univariate analysis**				
Trastuzumab +/- chemotherapy vs. untreated	HR 0.63<br>95% CI 0.33-1.19<br>*p*=0.154	HR 0.68<br>95% CI 0.51-0.89<br>*p*=0.004**	HR 1.21<br>95% CI 0.37-3.96<br>*p*=0.756	HR 0.50<br>95% CI 0.31-0.79<br>*p*=0.002**
Trastuzumab vs. untreated	HR 0.28<br>95% CI 0.09-0.95<br>*p*=0.041**	HR 0.55<br>95% CI 0.34-0.89<br>*p*=0.014**	HR 1.02<br>95% CI 0.20-5.27<br>*p*=0.981	HR 0.35<br>95% CI 0.14-0.90<br>*p*=0.027**
Combination vs. untreated	HR 0.88<br>95% CI 0.44-1.73<br>*p*=0.707	HR 0.71<br>95% CI 0.53-0.94<br>*p*=0.016**	HR 1.33<br>95% CI 0.36-4.95<br>*p*=0.673	HR 0.53<br>95% CI 0.33-0.86<br>*p*=0.0.01**
Combination vs. trastuzumab	HR 3.08<br>95% CI 0.88-10.83<br>*p*=0.079	HR 1.30<br>95% CI 0.80-2.09<br>*p*=0.287	HR 1.3<br>95% CI 0.24-7.11<br>*p*=0.761	HR 1.53<br>95% CI 0.60-3.94<br>*p*=0.375
Overall test on treatment effect	*p*=0.094	*p*=0.01**	*p*=0.906	*p*=0.007**
**Multivariate analysis**				
Trastuzumab +/- chemotherapy vs. untreated	HR 0.53<br>95% CI 0.26-1.09<br>*p*=0.084	HR 0.69<br>95% CI 0.52-0.92<br>*p*=0.012	N/A	N/A
Trastuzumab vs. untreated	HR 0.23<br>95% CI 0.06-0.84<br>*p*=0.026**	HR 0.62<br>95% CI 0.37-1.02<br>*p*=0.061		
Combination vs. untreated	HR 0.78<br>95% CI 0.35-1.73<br>*p*=0.544	HR 0.70<br>95% CI 0.52-0.95<br>*p*=0.023**		
Combination vs. trastuzumab	HR 3.36<br>95% CI 0.86-13.21<br>*p*=0.082	HR 1.14<br>95% CI 0.70-1.86<br>*p*=0.602		
Overall test on treatment effect	*p*=0.084	*p*=0.037**		

Notes: **Denotes statistical significance between comparator groups. Multivariate analysis using a Cox proportional hazard model was used to control for the following factors: age, race, ethnicity, BMI, hormone receptor status, tumor grade, histology type, T1 substage, BRCA mutation status, geographical region, functional status, and smoking history.

Abbreviations: CI = confidence interval, iDFS = invasive disease-free survival, OS = overall survival

Supplemental Table 2. Summary of iDFS and OS within our two- and three-arm comparisons for each T1 sub-categorization

	T1a iDFS	T1b iDFS	T1c iDFS	T1a OS	T1b OS	T1c OS
**Univariate analysis**						
Trastuzumab +/- chemotherapy vs. untreated	HR 0.63 95% CI 0.33-1.19 *p*=0.154	HR 0.64 95% CI 0.4-1.01 *p*=0.052	HR 0.69 95% CI 0.49-0.98 *p*=0.035**	HR 1.21 95% CI 0.37-3.96 *p*=0.756	HR 0.50 95% CI 0.2-1.25 *p*=0.133	HR 0.46 95% CI 0.27-0.79 *p*=0.004**
Trastuzumab vs. untreated	HR 0.28 95% CI 0.09-0.95 *p*=0.041**	HR 0.59 95% CI 0.25-1.39 *p*=0.226	HR 0.53 95% CI 0.3-0.96 *p*=0.036**	HR 1.02 95% CI 0.20-5.27 *p*=0.981	HR 1.01 95% CI 0.28-3.62 *p*=0.99	HR 0.16 95% CI 0.04-0.68 *p*=0.013**
Combination vs. untreated	HR 0.88 95% CI 0.44-1.73 *p*=0.707	HR 0.65 95% CI 0.4-1.05 *p*=0.079	HR 0.74 95% CI 0.52-1.05 *p*=0.089	HR 1.33 95% CI 0.36-4.95 *p*=0.673	HR 0.39 95% CI 0.13-1.12 *p*=0.079	HR 0.54 95% CI 0.13-1.12 *p*=0.026**
Combination vs. trastuzumab	HR 3.08 95% CI 0.88-10.83 *p*=0.079	HR 1.1 95% CI 0.46-2.66 *p*=0.827	HR 1.38 95% CI 0.78-2.44 *p*=0.267	HR 1.3 95% CI 0.24-7.11 *p*=0.761	HR 0.38 95% CI 0.09-1.61 *p*=0.191	HR 3.32 95% CI 0.79-13.99 *p*=0.102
Overall test on treatment effect	*p*=0.094	*p*=0.149	*p*=0.063	*p*=0.906	*p*=0.167	*p*=0.005**
**Multivariate analysis**						
Trastuzumab +/- chemotherapy vs. untreated	HR 0.53 95% CI 0.26-1.09 *p*=0.084	HR 0.68 95% CI 0.41-1.15 *p*=0.155	HR 0.65 95% CI 0.45-0.94 *p*=0.02**	N/A	N/A	
Trastuzumab vs. untreated	HR 0.23 95% CI 0.06-0.84 *p*=0.026**	HR 0.60 95% CI 0.23-1.55 *p*=0.295	HR 0.57 95% CI 0.3-1.05 *p*=0.071			
Combination vs. untreated	HR 0.78 95% CI 0.35-1.73 *p*=0.544	HR 0.70 95% CI 0.41-1.21 *p*=0.204	HR 0.67 95% CI 0.46-0.98 *p*=0.039**			
Combination vs. trastuzumab	HR 3.36 95% CI 0.86-13.21 *p*=0.082	HR 1.16 95% CI 0.45-2.99 *p*=0.752	HR 1.19 95% CI 0.65-2.18 *p*=0.576			
Overall test on treatment effect	*p*=0.084	*p*=0.348	*p*=0.06			

Notes: **Denotes statistical significance between comparator groups. Multivariate analysis using a Cox proportional hazard model was used to control for the following factors: age, race, ethnicity, BMI, hormone receptor status, tumor grade, histology type, T1 substage, BRCA mutation status, geographical region, functional status, and smoking history.

Abbreviations: CI = confidence interval, iDFS = invasive disease-free survival, OS = overall survival

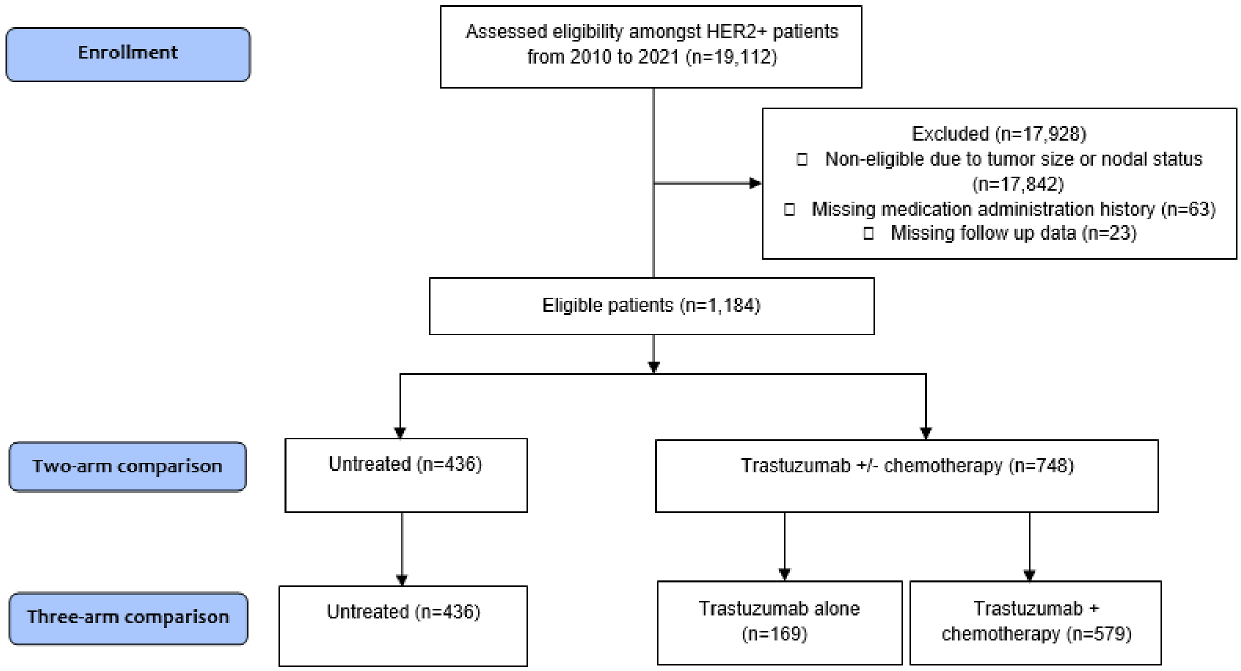

Supplemental Figure 1. CONSORT diagram highlighting the patient selection process for this retrospective study

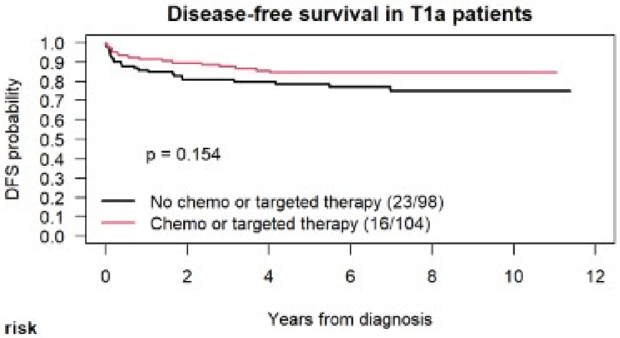

No. at risk						
No chemo or targeted therapy	98	79	74	50	29	7
Chemo or targeted therapy	104	93	80	46	22	7

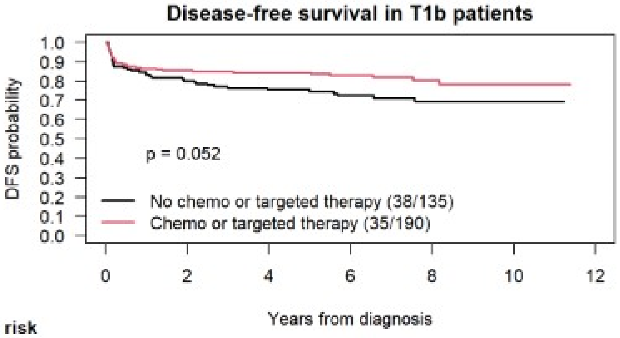

No. at risk						
No chemo or targeted therapy	135	108	93	59	31	10
Chemo or targeted therapy	190	162	144	91	44	11

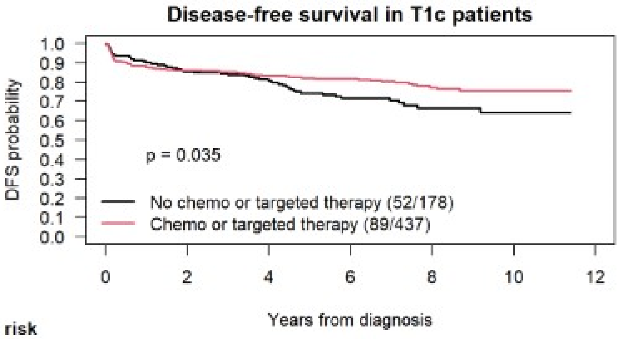

No. at risk						
No chemo or targeted therapy	178	149	135	78	44	15
Chemo or targeted therapy	437	373	334	227	105	31

Abbreviations: iDFS = invasive disease-free survival, chemo = chemotherapy

Supplemental Figure 2. Univariate analysis of iDFS among T1 sub-categorizations in our two-group comparison of locoregional therapy alone versus trastuzumab with or without chemotherapy

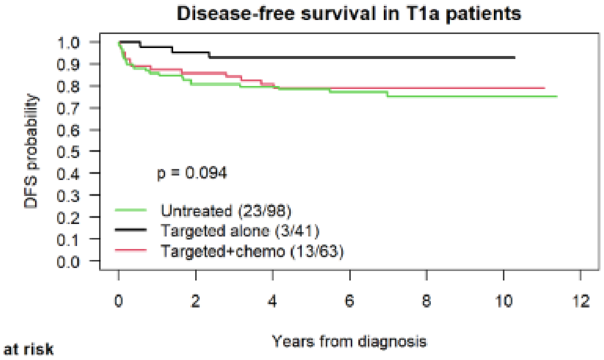

No. at risk

Untreated	98	79	74	50	29	7
Targeted alone	41	39	35	20	8	3
Targeted_chemo	63	54	45	26	14	4

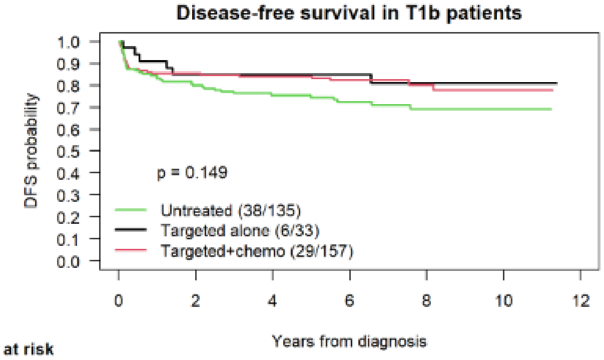

No. at risk

Untreated	135	108	93	59	31	10
Targeted alone	33	28	27	22	9	3
Targeted_chemo	157	134	117	69	35	8

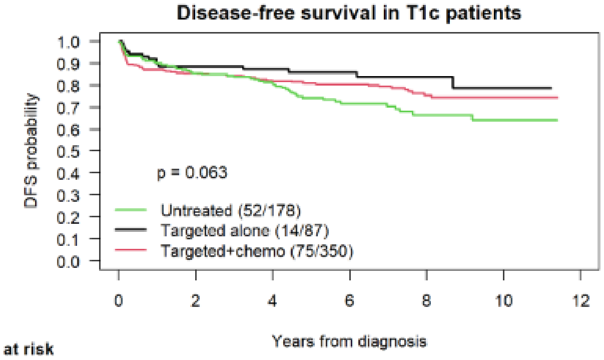

No. at risk

Untreated	178	149	135	78	44	15
Targeted alone	87	76	68	42	26	9
Targeted_chemo	350	297	266	185	79	22

Abbreviations: iDFS = invasive disease-free survival, chemo = chemotherapy

Supplemental Figure 3. Univariate analysis of iDFS among T1 sub-categorizations in our three-group comparison of locoregional therapy alone versus trastuzumab with or without chemotherapy

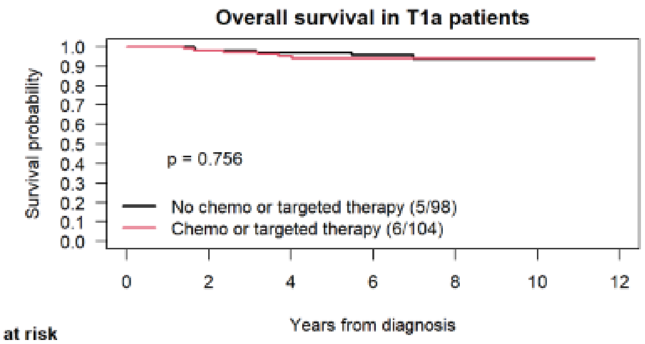

No. at risk						
No chemo or targeted therapy	98	96	89	62	32	8
Chemo or targeted therapy	104	102	87	52	24	9

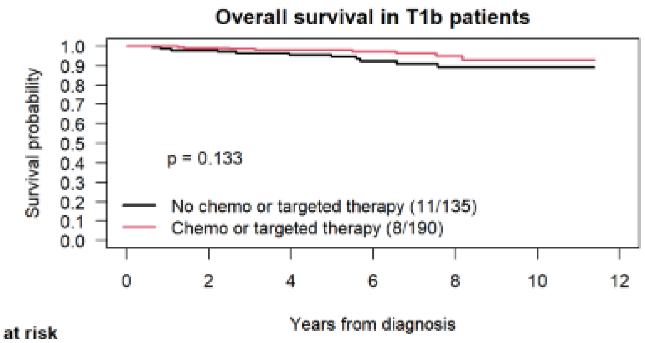

No. at risk						
No chemo or targeted therapy	135	132	113	72	39	15
Chemo or targeted therapy	190	188	167	111	56	16

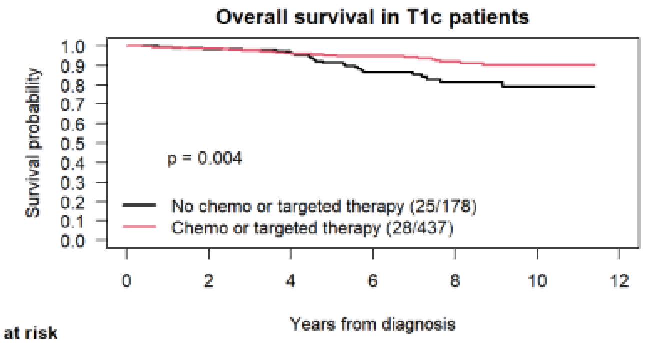

No. at risk						
No chemo or targeted therapy	178	172	162	98	53	19
Chemo or targeted therapy	437	426	379	258	123	37

Abbreviations: OS = overall survival, chemo = chemotherapy

Supplemental Figure 4. Univariate analysis of OS among T1 sub-categorizations in our two-group comparison of locoregional therapy alone versus trastuzumab with or without chemotherapy

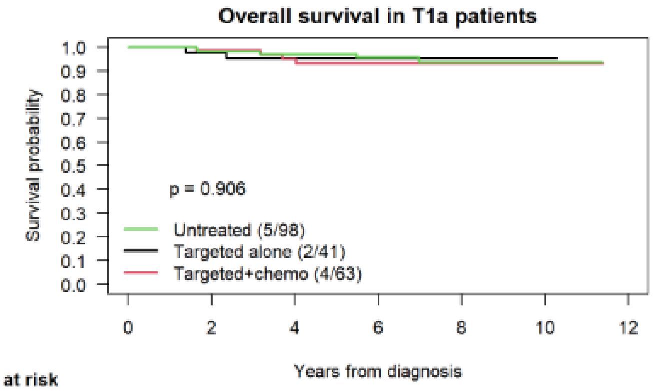

No. at risk						
Untreated	98	96	89	62	32	8
Targeted alone	41	40	35	20	8	3
Targeted_chemo	63	62	52	32	16	6

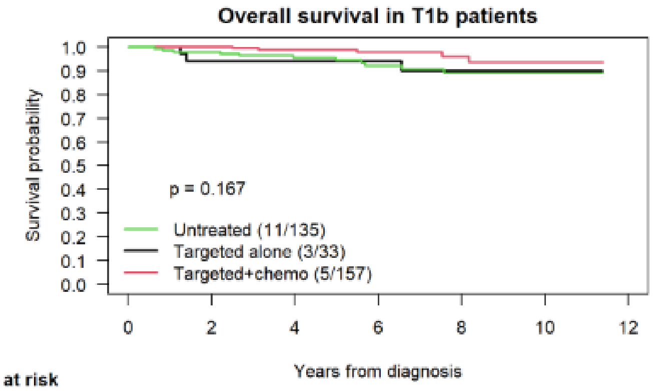

No. at risk						
Untreated	135	132	113	72	39	15
Targeted alone	33	31	30	25	11	3
Targeted_chemo	157	157	137	86	45	13

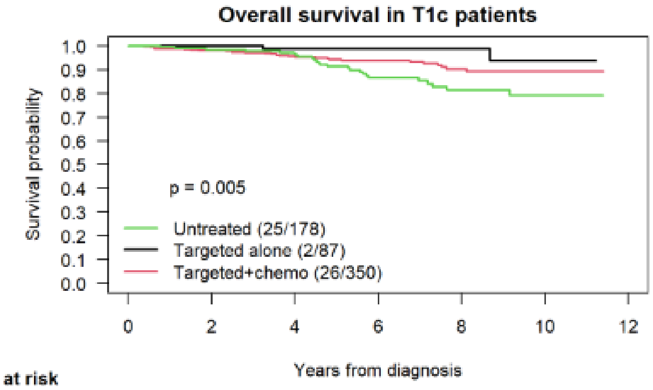

No. at risk						
Untreated	178	172	162	98	53	19
Targeted alone	87	86	76	47	29	10
Targeted_chemo	350	340	303	211	94	27

Abbreviations: OS = overall survival, chemo = chemotherapy

Supplemental Figure 5. Univariate analysis of OS among T1 sub-categorizations in our three-group comparison of locoregional therapy alone versus trastuzumab with or without chemotherapy
